# Differential effect of ethanol intoxication on peripheral markers of cerebral injury in murine blunt traumatic brain injury

**DOI:** 10.1093/burnst/tkab027

**Published:** 2021-09-30

**Authors:** Zhenghui Li, Jin Zhang, Steffen Halbgebauer, Akila Chandrasekar, Rida Rehman, Albert Ludolph, Tobias Boeckers, Markus Huber-Lang, Markus Otto, Francesco Roselli, Florian olde Heuvel

**Affiliations:** Department of Neurology, Ulm University, ZBMF - Helmholtzstrasse 8/1, 89081 Ulm, Germany; Department of Neurosurgery, Kaifeng Central Hospital, Kaifeng, 475000 Henan China; Department of Neurology, Ulm University, ZBMF - Helmholtzstrasse 8/1, 89081 Ulm, Germany; Department of Neurology, Ulm University, ZBMF - Helmholtzstrasse 8/1, 89081 Ulm, Germany; Department of Neurology, Ulm University, ZBMF - Helmholtzstrasse 8/1, 89081 Ulm, Germany; Department of Neurology, Ulm University, ZBMF - Helmholtzstrasse 8/1, 89081 Ulm, Germany; Department of Neurology, Ulm University, ZBMF - Helmholtzstrasse 8/1, 89081 Ulm, Germany; German Center for Neurodegenerative Diseases (DZNE), Ulm, Germany; German Center for Neurodegenerative Diseases (DZNE), Ulm, Germany; Institute of Anatomy and Cell Biology, Ulm University, Ulm, Germany, M24, Albert-Einstein Allee 11, 89081 Ulm, Germany; Institute of Clinical and Experimental Trauma-Immunology, University Hospital, ZBMF - Helmhotzstrasse 8/1, 89081 Ulm, Germany; Department of Neurology, Ulm University, ZBMF - Helmholtzstrasse 8/1, 89081 Ulm, Germany; Department of Neurology, Ulm University, ZBMF - Helmholtzstrasse 8/1, 89081 Ulm, Germany; German Center for Neurodegenerative Diseases (DZNE), Ulm, Germany; Department of Neurology, Ulm University, ZBMF - Helmholtzstrasse 8/1, 89081 Ulm, Germany

**Keywords:** Traumatic brain injury, Ethanol, Biomarkers, neurofilament light, neuron-specific enolase, S100B, Claudin-5

## Abstract

**Background:**

Blood-based biomarkers have proven to be a reliable measure of the severity and outcome of traumatic brain injury (TBI) in both murine models and patients. In particular, neuron-specific enolase (NSE), neurofilament light (NFL) and S100 beta (S100B) have been investigated in the clinical setting post-injury. Ethanol intoxication (EI) remains a significant comorbidity in TBI, with 30–40% of patients having a positive blood alcohol concentration post-TBI. The effect of ethanol on blood-based biomarkers for the prognosis and diagnosis of TBI remains unclear. In this study, we investigated the effect of EI on NSE, NFL and S100B and their correlation with blood–brain barrier integrity in a murine model of TBI.

**Methods:**

We used ultra-sensitive single-molecule array technology and enzyme-linked immunosorbent assay methods to measure NFL, NSE, S100B and claudin-5 concentrations in plasma 3 hours post-TBI.

**Results:**

We showed that NFL, NSE and S100B were increased at 3 hours post-TBI. Interestingly, ethanol blood concentrations showed an inverse correlation with NSE but not with NFL or S100B. Claudin-5 levels were increased post-injury but no difference was detected compared to ethanol pretreatment. The increase in claudin-5 post-TBI was correlated with NFL but not with NSE or S100B.

**Conclusions:**

Ethanol induces an effect on biomarker release in the bloodstream that is different from TBI not influenced by alcohol. This could be the basis of investigations into humans.

Highlights

- Experimental traumatic brain injury in mice resulted in an increase in plasma concentrations of NFL, NSE and S100B.
- Ethanol blood concentrations showed an inverse correlation with plasma NSE but not NFL or S100B.
- NFL concentrations post-injury showed a correlation with blood–brain barrier disruption, as shown by plasma claudin-5 concentrations.

## Background

Blood-based biomarkers serve as promising candidates for defining neuronal, glial and vascular damage in cases of traumatic brain injury (TBI) [1, 2]. In particular, serum levels of the neuronal cytoplasmic enzyme neuron-specific enolase (NSE) have been found to be increased in head trauma in patients [3, 4]; its elevation was inversely correlated with Glasgow Coma Scale score [5] and predictive of long-term connectivity disturbances [6] and overall outcome in cases of TBI [4]. Potentially complementary to NSE, the levels of the intermediate filament that is highly enriched in axons, neurofilament light (NFL)-chain, have recently been shown to be upregulated in blood and cerebrospinal fluid (CSF) samples from TBI patients [7, 8] as well as in samples from patients with neurodegeneration [9, 10]. Likewise, levels of astrocyte-specific S100B have been shown to be upregulated in TBI [11] and appear to be predictive of substantial anatomical damage [12]. Plasma biomarkers of neuronal damage have only been recently applied to murine models of TBI as proxies of neuronal, axonal and glial damage (NSE, NFL and S100B, respectively) [13–16].

The purpose of plasma biomarkers in clinical practice requires clarification of how they may be affected, and interpretation of their levels confounded, by comorbidities and other factors concomitant to TBI. Ethanol intoxication (EI) is, to date, the most common comorbidity in TBI, with 30–40% of patients showing a positive blood alcohol concentration (BAC) [17], amounting to more than 1 million patients being admitted with concomitant TBI and EI every year in the US (out of a total of approximately 3 million) [18]. Clinical research has produced conflicting results around whether EI may lead to false-positive elevation of the S100B glial damage marker [19–21]. It is currently unclear whether ethanol may have similar effects on markers of neuronal damage, such as NSE and NFL. Furthermore, the issue of whether EI co-occurring with TBI may be detrimental or beneficial is controversial, as positive BAC at the time of TBI has been associated with a better prognosis in some studies [22–26] but not in others [27, 28]. In experimental TBI, EI decreases TBI-induced neuroinflammation, neuronal loss and behavioral disturbances [29–32], although not in all studies [33, 34]. It is unclear whether any beneficial effect of EI on plasma biomarkers of neuronal or glial damage occurring in cases of TBI can be detected. In the present study, we explored the effects of EI on the elevation of plasma biomarkers (NSE, NFL, S100B and the marker of blood–brain barrier (BBB) integrity, claudin-5) associated with TBI in a *post hoc* analysis of murine samples from previous studies. Our model involves the administration of a single, comparatively large dose of ethanol to young male mice before TBI; this model mimics the high prevalence of TBI injury in young male adults especially over weekends (drink-and-drive model) [35–37].

We found an elevation of the 4 plasma biomarkers in TBI and that only NSE levels were inversely correlated with BAC; interestingly, EI alone was sufficient to elevate S100B to a level comparable to what is seen in TBI.

## Methods

### Animals, TBI model and ethanol treatment

This was a *post hoc*, hypothesis-driven analysis of blood samples obtained in the context of previous studies [29–32]; the investigation of these samples has never been reported and was undertaken in accordance with the 3R principle (replacement, reducemeent and refinement), that is, to reduce the number of mice used in animal experiments and to maximize the scientific output from animal sacrifice. All samples were obtained during 2016–2017 and stored at −80°C until use. Experimental procedures were performed by the same operators.

These experiments were approved by the Regierungspräsidium Tübingen under animal license number 1222, with successive integrations, and by the Ulm University Animal Experimentation Oversight Committee.

Male B6-SJL mice were bred locally (Ulm University) under standard husbandry conditions (24°C, 60–80% humidity, 12 hour light/dark cycle and *ad libitum* access to food and water). Male mice aged 60–90 days were used, in agreement with overall epidemiological trends of TBI in human patients [35–37]. Experimental TBI was performed as previously reported [29–32]. Briefly, after administration of buprenorphine (0.1 mg/kg by subcutaneous injection) and under sevoflurane anesthesia (2.5% in 97.5% O_2_), mice aged 60–90 days were subject to closed weight-drop TBI. Animals were then manually positioned in the weight-drop apparatus and TBI was delivered by a 333 g impactor free-falling from a 2-cm distance, targeting the parietal bone [38]. Immediately after the experimental TBI, animals were administered 100% O_2_ and monitored for the apnea time. For sham surgery, mice were subjected to the same procedures and treatments (anesthesia, skin opening and closure, handling, positioning in the TBI apparatus) but no trauma was delivered. Ethanol was administered as previously described [39, 40]: the ethanol solution was obtained by diluting synthesis-grade 100% ethanol in 0.9% saline, giving a final dilution of 32% volume/volume (32 μL of 100% ethanol and μL microliters of saline). Since the density of ethanol is approximately 0.78 g/mL, 100 μL of the 32% solution contained 25 μg of ethanol. In order to administer a set dose of 5 g/kg (or 5 μg/g) for a mouse in the range of 20–25 g, a volume of 400–500 μL was administered by oral gavage 30 minutes prior to TBI. Four experimental groups were considered: saline administered and subjected to sham surgery (saline–sham (SS), n = 8); saline administered and subjected to TBI (saline–TBI (ST), n = 24); ethanol administered and subjected to sham surgery (ethanol–sham (ES), n = 14); and ethanol administered and subjected to TBI (ethanol–TBI (ST), n = 17).

**Perfusion–fixation and free-floating immunofluorescence staining** Mice were sacrificed by intracardiac perfusion at 3-hour and 7-day timepoints as previously reported [29]. Briefly, mice were perfused with 1 mL/g PBS and 2.5 mL/g 4% paraformaldehyde (PFA) in phosphate buffered saline (PBS) at a rate of 2.5 mL/minute. The brain was dissected and post-fixed in 4% PFA overnight at 4°C, cryoprotected in 30% sucrose and embedded in Tissue-Tek, optimal cutting temperature (OCT, Sakura, Germany). Cryostat brain sections of 40-μm thickness were cut from the site of injury and subjected to fluorescence immunohistochemistry according to previously reported procedures [32]. Briefly, sections were washed in PBS twice for 20 minutes, blocked for 2 hours at room temperature (RT) in blocking buffer (3% Bovine serum albumin (BSA) and 0.3% triton in PBS) and incubated with mouse anti-NeuN (1:300; Millipore, Germany) in blocking buffer for 48 hours at 4°C. Sections were washed 3 times for 30 minutes in PBS at room temperature and incubated with donkey anti-mouse (1:500; Invitrogen, Germany) diluted in blocking buffer for 2 hours at RT followed by another set of 3 30-minute washes in PBS at RT. Sections were dried and mounted with fluorogold prolong antifade mounting medium (Invitrogen, Germany).

**Confocal imaging and image analysis** neuronal nuclear protein (NeuN) immunofluorescence staining was acquired using an LSM-710 (Carl Zeiss AG) microscope fitted with a ×20 air objective with optical thickness fitted to the optimum value. For overview images, ×20 objective 3 × 5 image tiles were acquired. All images were acquired at 1024 × 1024 pixels resolution and 16-bit depth. Acquisition parameters were set to avoid over- or under-saturation and kept constant for each experimental set. For image analysis, images were loaded in ImageJ (Public domain, NIH, USA) and neuronal density was calculated by counting NeuN-positive cells in a fixed area in the middle of the impact site (core) and a fixed area at a fixed distance away from the injury site (penumbra).

### Blood sampling and plasma preparation

Three hours after trauma, animals were subjected to xylazine/ketamine terminal anesthesia. Blood was collected by right ventricular puncture using a 1-mL syringe equipped with a 24-Gauge (24-G) needle and quickly transferred to a vial containing Ethylenediaminetetraacetic acid (EDTA) as anticoagulant. From each mouse, 400–500 μL of blood (200–300 μL of plasma) was collected. Plasma samples were prepared by centrifuging the EDTA vials for 5 minutes at 800 *g* at 4°C; the supernatant was collected and centrifuged again for 2 minutes at 13,000 *g* at 4°C. Plasma was aliquoted and stored at −80°C until use.

### Blood ethanol assay

The ethanol blood assay was performed according to the manufacturer’s instructions (Abcam, United Kingdom). Briefly, plasma was diluted in double distilled H_2_O (ddH_2_O) (1:500 for ethanol samples and 1:10 for saline samples). Samples were added to a 96-well plate and a master mix of ethanol probe, ethanol enzyme mix and ethanol assay buffer was added and incubated for 30 minutes at 37°C. The optical density (OD) of 450 nm was measured using colorimetric detection (Fluostar Optima, BMG Labtech, Germany) and concentrations were calculated with the standard curve.

### Single-molecule array determination of plasma NFL

Single-molecule array quantification of plasma NFL chain (Quanterix, USA) was performed as previously reported [41] and in accordance with the manufacturer’s instructions. Mouse plasma (5 μL) was diluted 1:20 before the assay.

### Enzyme-linked immunosorbent assay determination of plasma NSE, S100B and claudin-5

Enzyme-linked immuno-sorbent assays (ELISAs) for NSE, claudin-5 and S100 beta (S100B) were performed according to the instructions of each manufacturer (mouse ENO2/NSE (CLIA) ELISA Kit, LSbio, USA; mouse claudin-5 ELISA Kit, Cusabio, USA; mouse S100 Beta ELISA Kit, LSbio, USA, respectively). Briefly, 100 μL of diluted samples (NSE: 1:10; claudin-5: 1:2; S100B: 1:2, diluted in sample diluent) or standards were added to the well and incubated for 90 minutes at 37°C, followed by aspiration of the liquid and directly followed by adding 100 μL of 1× biotinylated detection antibody working solution for 1 hour at 37°C. The wells were aspirated and then washed 3 times by adding 350 μL of washing buffer to each well for 2 minutes at RT, after which 100 μL of 1× Horseradisch peroxidase (HRP) conjugate was added to the well and incubated for 30 minutes at 37°C. The wells were aspirated and washed 5 times with 350 μL of washing buffer for 2 minutes each. Finally, 100 μL of working substrate solution was added to each well and incubated for 5 minutes at 37°C (for the claudin-5 ELISA, 50 μL of stop solution was added), after which the relative light units (for NSE) or OD at 450 nm (for claudin-5 and S100B) were measured by using a microplate luminometer (Luminoskan Ascent, Thermo Scientific, Germany). Concentrations were calculated according to the standard curve.

### Data analysis

Statistical analysis was performed using GraphPad Prism version 8 software (Graphpad software, USA). The Shapiro–Wilk test was performed to test all groups for normality. Grouped analysis for neuronal density was performed using two-way analysis of variance (ANOVA) with Tukey’s multiple correction. Grouped analysis for the ELISA and single-molecule array (SIMOA) data was performed using ANOVA with Tukey’s multiple correction or the Kruskal–Wallis test with Dunn’s multiple correction, depending on normality. Pearson’s *r* correlation assay or Spearman’s *r* correlation assay, depending on normality, were performed between treatment groups and different analytes to assess the relationships. Analysis of covariance (ANCOVA) was undertaken by comparing linear regression slopes of treatment groups with Tukey’s multiple correction. Data are depicted in graphs as medians, 25th to 75th percentiles (box), minimum to maximum (whiskers) or correlation with linear regression. Data in the text are depicted as median (minimum to maximum). Statistical significance was set at *p* < 0.05.

## Results

### Ethanol pretreatment resulted in decreased neuronal loss post-TBI

**Figure 1.**
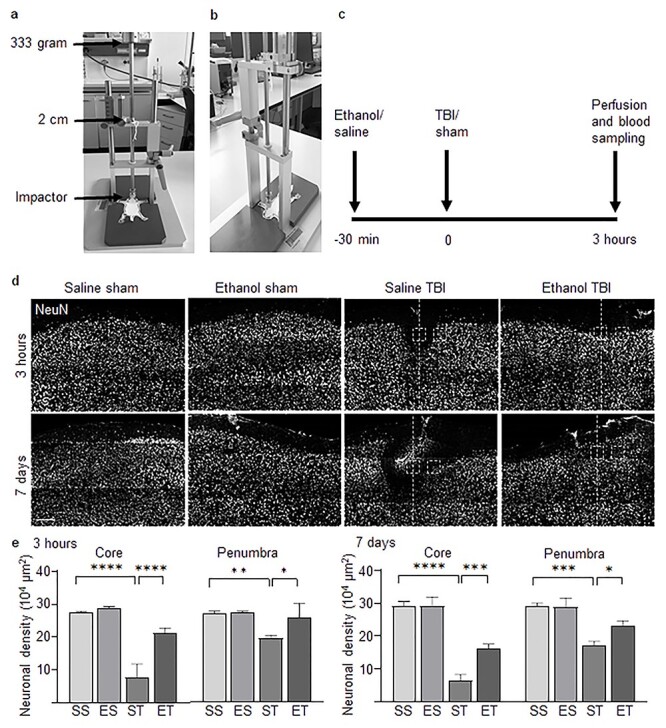
Ethanol pretreatment results in decreased neuronal loss after traumatic brain injury (TBI). Immunofluorescence staining of neuronal nuclear protein (NeuN) 3 hours and 7 days after TBI. Four treatment groups were used: saline–sham (SS), ethanol–sham (ES), saline–TBI (ST) and ethanol–TBI (ET). **(a, b)** Picture of the blunt weight-drop model used in this study, depicting the impactor, distance and weight. **(c)** Workflow of ethanol/saline administration, TBI/sham operation and collection of blood post-surgery. **(d, e)** NeuN immunofluorescence staining reveals neuronal density at the injury site 3 hours and 7 days after TBI. Analysis of NeuN density was performed in the core of the injury (fixed area immediately under the impact site) and in the penumbra (fixed area within a fixed distance from the core). The TBI groups showed a significant decrease in neuronal integrity at both 3 hours (*p* < 0.0001 for core, *p* < 0.01 for penumbra) and 7 days (*p* < 0.0001 for core, *p* < 0.001 for penumbra) post-injury. Ethanol pretreatment resulted in a significant decrease in neuronal loss at both 3 hours (*p* < 0.0001 for core, *p* < 0.05 for penumbra) and 7 days (*p* < 0.001 for core, *p* < 0.05) after TBI. Histograms show mean ± standard deviation. Group sizes: SS, n = 4; ES, n = 4; ST, n = 4; ET, n = 4. Scale bar: 200 μm. ^*^p < 0.05, ^**^p < 0.01, ^***^p < 0.001, ^****^p < 0.0001

First, we assessed the neuronal damage in the cortex directly under the injury site 3 hours and 7 days post-TBI. For this, mice were pretreated with saline or ethanol (5 g/kg) 30 minutes before either TBI or sham surgery. Four experimental groups were therefore considered: SS, ES, ST and ET (n = 4). Mice were perfused at 3-hour and 7-day time points and cryosections were stained with NeuN to establish neuronal density. The neuronal densities in a fixed area in the injury site (core) and a fixed area away from the injury site (penumbra) were counted. TBI resulted in a significant decrease in NeuN density in both the core and penumbra compared to SS at 3 hours (28 ± 1 *vs* 8 ± 4 per 10^4^ μm^2^; SS *vs* ST; *p* < 0.0001 for core; 28 ± 2 *vs* 17 ± 6 per 10^4^ μm^2^; SS *vs* ST; *p* < 0.01 for penumbra; Figure 1d, e) and 7 days (29 ± 2 *vs* 6 ± 2 per 10^4^ μm^2^; SS *vs* ST; *p* < 0.0001 for core; 29 ± 1 *vs* 19 ± 2 per 10^4^ μm^2^; SS *vs* ST; *p* < 0.001 for penumbra; Figure 1d, e) post-injury. Ethanol pretreatment resulted in a significant decrease in neuronal loss compared with TBI, in both core and penumbra, at 3 hours (8 ± 4 *vs* 21 ± 4 per 10^4^ μm^2^; ST *vs* ET; *p* < 0.0001 for core; 17 ± 6 *vs* 26 ± 5 per 10^4^ μm^2^; ST *vs* ET; *p* < 0.05 for penumbra; Figure 1d, e) and 7 days (6 ± 2 *vs* 16 ± 2 per 10^4^ μm^2^; ST *vs* ET; *p* < 0.001 for core; 19 ± 2 *vs* 24 ± 2 per 10^4^ μm^2^; ST *vs* ET; *p* < 0.05 for penumbra; Figure 1d, e).

### Blood ethanol concentration is not affected by TBI after oral binge

**Figure 2.**
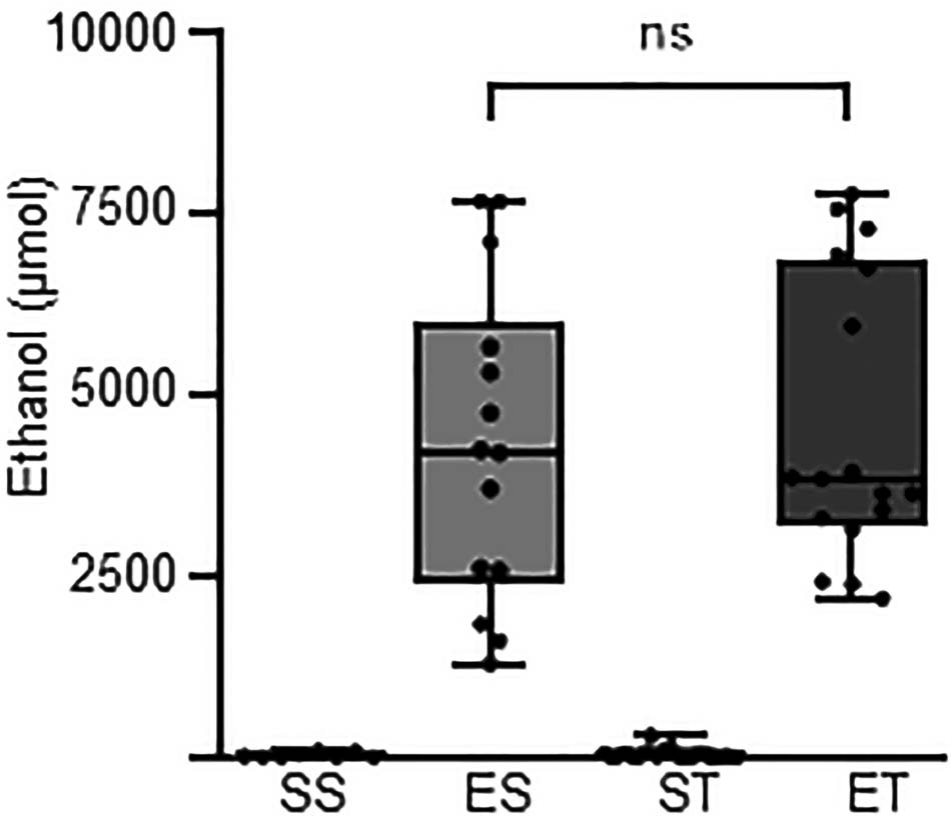
Traumatic brain injury (TBI) does not affect ethanol concentration after binge administration. Blood alcohol concentration in blood plasma 3 hours after TBI. Four treatment groups were used: saline–sham (SS), ethanol–sham (ES), saline–TBI (ST) and ethanol–TBI (ET). Ethanol treatment showed a significant difference between treatment groups (*p* < 0.0001). *Post hoc* analysis revealed a significant difference between SS and ES (*p* < 0.0003) and between ST and ET (*p* < 0.0001). However, ES and ET showed no difference (*p* > 0.9999). Boxplots represent median value, 25th to 75th percentile (box) and minimum to maximum (whiskers), including individual data points. Group sizes: SS, n = 8; ES, n = 14; ST, n = 24; ET, n = 17. ^*^p < 0.05, ^**^p < 0.01, ^***^p < 0.001, ^****^p < 0.0001. *ns* not significant

Then, we established the concentration of ethanol in plasma upon single oral binge with or without concomitant TBI. For this, mice were pretreated with saline or ethanol (5 g/kg) 30 minutes before either TBI or sham surgery; ethanol concentrations were measured at 3 hours after the trauma. Four experimental groups were therefore considered: SS (n = 8), ES (n = 14), ST (n = 24) and ET (n = 17). The Kruskal–Wallis test showed a significant effect on ethanol concentrations between treatment groups (*p* < 0.0001; Figure 2). The *post hoc* comparison (Dunn’s corrected) revealed a significant difference, predictably, between SS and ES (8.9 (0 –84.0) μmol *vs* 4208.9 (1279.1–7661.6) μmol; *p* = 0.0003; Figure 1d) and between ST and ET (13.5 (0–291.0) μmol *vs* 3832.1 (2186.5–7753.9) μmol; *p* < 0.0001; Figure 2), but ES and ET showed comparable plasma ethanol concentrations (*p* > 0.9999; Figure 2). These findings suggest that TBI does not, per se, affect the clearance of ethanol after oral binge.

### EI blunts NSE but not NFL or S100B upregulation after TBI

**Figure 3.**
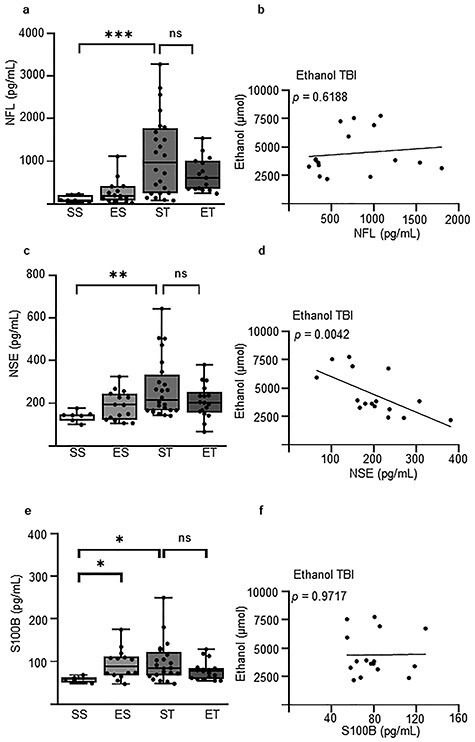
Ethanol is inversely correlated with neuron-specific enolase (NSE) levels but not with neurofilament light (NFL) and S100 beta (S100B) levels post-traumatic brain injury (TBI). NFL, NSE and S100B concentrations were assessed 3 hours post-TBI. Four treatment groups were used: saline–sham (SS), ethanol–sham (ES), saline–TBI (ST) and ethanol–TBI (ET). **(a)** Treatment groups showed significant differences in NFL concentration (*p* < 0.0001). *Post hoc* analysis showed a significant difference between SS and ST (*p* = 0.0005) but no difference between ST and ET (*p* > 0.9999). **(b)** Correlation analysis showed no significant relationship between NFL and ethanol in the ET group (*p* = 0.6188). **(c)** NSE concentration showed significant differences between treatment groups (*p* = 0.0064). *Post hoc* analysis revealed a significant difference between SS and ST (*p* = 0.0057), but not between ST and ET (*p* > 0.9999). **(d)** Correlation analysis revealed a significant inverse correlation between NSE and ethanol in the ET group (*p* = 0.0042). **(e)** S100B concentration showed significant differences between treatment groups (*p* = 0.0282). *Post hoc* analysis revealed a significant difference between SS and ES (*p* = 0.0451) and between SS and ST (*p* = 0.0267), but not between ST and ET (*p* > 0.9999). **(f)** Correlation analysis revealed no significant correlation between S100B and ethanol in the ET group (*p* = 0.9717). Boxplots represent median value, 25th to 75th percentile (box) and minimum to maximum (whiskers), including individual data points. Correlation with linear regression is shown. Group sizes: SS, n = 8; ES, n = 14; ST, n = 24; ET, n = 17. ^*^p < 0.05, ^**^p < 0.01, ^***^p < 0.001, ^****^p < 0.0001. *ns* not significant

Next, we set out to investigate the effect of EI on 2 plasma biomarkers of neuronal injury (NSE and NFL) and one plasma biomarker of glial injury (S100B) after TBI. For the NFL assessment, the Kruskal–Wallis test revealed a significant effect between treatment groups (*p* < 0.0001; Figure 3a). The *post hoc* comparison (Dunn’s corrected) showed that ethanol alone, in the absence of trauma, did not affect the NFL concentrations in plasma (SS: 78.3 (35.8–226.2) pg/ml *vs* ES: 190.3 (20.9–1112.3) pg/ml; *p* > 0.9999; Figure 3a). There was a significant upregulation in the TBI group in comparison with baseline (SS: 78.3 (35.8–226.2) pg/ml *vs* ST: 969.4 (76.7–3276) pg/ml; *p* = 0.0005; Figure 3a). The group that received ethanol treatment prior to TBI showed only a trend towards lower concentrations in comparison with the ST group (ST: 969.4 (76.7–3276) pg/ml *vs* ET: 608.8 (238.5–1541.2) pg/ml; *p* > 0.9999; Figure 3a). Interestingly, concentrations of plasma ethanol were completely uncorrelated with NFL concentrations in the ET group (*p* = 0.6188; Figure 3b).

Likewise, the Kruskal–Wallis Test, when applied to the NSE dataset, revealed a significant effect between treatment groups (*p* = 0.0064; Figure 3c). The *post hoc* comparison (Dunn’s corrected) revealed that ethanol alone showed no effect on NSE plasma concentrations (SS: 144.0 (98.6–291.5) *vs* ES: 191.9 (106.0–323.9); *p* = 0.6143; Figure 3c). There was a significant effect of TBI in comparison with sham (SS: 144.0 (98.6–291.5) *vs* ST: 215.7 (140.8–644.1); *p* = 0.0057; Figure 3c). However, ethanol treatment before TBI showed no significant difference between TBI on its own (ST: 215.7 (140.8–644.1) pg/ml *vs* ET: 202.0 (65.9–380.5) pg/ml; *p* > 0.9999; Figure 3c), but a significant inverse correlation was found between ethanol concentrations and NSE concentrations in the ET group (*p* = 0.0042; Figure 3d).

Lastly, for the levels of S100B, the Kruskal–Wallis test revealed a significant effect between treatment groups (*p* < 0.0282; Figure 3e). The *post hoc* comparison (Dunn’s corrected) revealed a significant increase already in the ES group compared with the SS group (SS: 57.6 (48.8–68.1) pg/ml *vs* ES: 104.7 (47.0–255.5) pg/ml; *p* < 0.0451; Figure 2e). TBI resulted in an increase of plasma S100B compared with the sham group (SS: 57.6 (48.8–68.1) pg/ml *vs* ST: 84.6 (47.6–249.8) pg/ml; *p* < 0.0267; Figure 3e) but, ethanol did not alter the S100B concentration after TBI (ST: 84.6 (47.6–249.8) pg/ml *vs* ET: 77.4 (54.7–128.6) pg/ml; *p* > 0.9999; Figure 3e). Concentrations of plasma ethanol were not correlated with S100B concentrations in the ET group (*p* = 0.9717; Figure 3f).

Thus, the amount of blood ethanol is inversely correlated with NSE levels in TBI. On the other hand, ethanol alone is sufficient to induce an elevation in S100B without co-occurring trauma.

### EI does not affect TBI-induced vascular disruption

**Figure 4.**
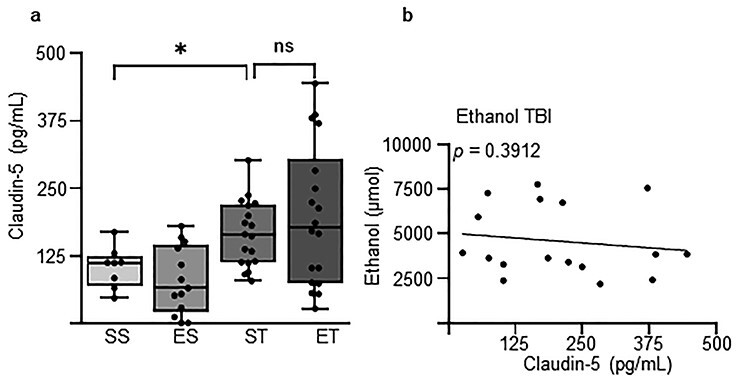
Blood–brain barrier (BBB) disruption post-TBI is unaffected by ethanol. Concentration of claudin-5 was assessed in the blood 3 hours post-traumatic brain injury (TBI) to investigate BBB disruption using 4 treatment groups: saline–sham (SS), ethanol–sham (ES), saline–TBI (ST) and ethanol–TBI (ET). **(a)** Treatment groups revealed significant differences in claudin-5 concentration (*p* = 0.0024). *Post hoc* analysis revealed significant differences between SS and ST (*p* = 0.0473) but no difference between ST and ET (*p* = 0.6788). **(b)** Correlation analysis between claudin-5 and ethanol showed no difference in the ET group (*p* = 0.3912). Boxplots represent median value, 25th to 75th percentile (box) and minimum to maximum (whiskers), including individual data points. Correlation with linear regression is shown. Group sizes: SS, n = 8; ES, n = 14; ST, n = 24; ET, n = 17. ^*^p < 0.05, ^**^p < 0.01, ^***^p < 0.001, ^****^p < 0.0001. *ns* not significant

Furthermore, we sought to identify whether ethanol modified the disruption of the BBB caused by TBI, since such disruption may not only contribute to the pathophysiology of TBI [42] but also affect the plasma concentrations of NSE, NFL or S100B [43]. We used plasma claudin-5 levels as a proxy of vascular involvement and BBB disruption [44, 45]. ANOVA revealed a significant difference between treatment groups (*F* = 5.458; *p* = 0.0024; Figure 4a). The *post hoc* analysis (Tukey’s corrected) showed no significant effect of ethanol by itself (SS: 111.8 (46.1–168.7) pg/ml *vs* ES: 64.8 (0–180.0) pg/ml; *p* = 0.9241; Figure 4a) but a significant increase caused by TBI (SS: 111.8 (46.1–168.7) pg/ml *vs* ST: 161.2 (77.9–237.0) pg/ml; *p* = 0.0473; Figure 4a) that was not mitigated by concomitant EI (ST: 161.2 (77.9–237.0) pg/ml *vs* ET: 178.0 (26.4–444.7) pg/ml; *p* = 0.6788; Figure 4a). Likewise, there was no significant correlation between the concentrations of ethanol and the concentrations of claudin-5 in the plasma (*p* = 0.3912; Figure 4b).

**Table 1:** Plasma neurofilament light (NFL), neuron-specific enolase (NSE) and S100 beta (S100B) concentrations do not correlate post traumatic brain injury. Correlation tables are shown, with both *R*^2^ and *p* values (if data are normally distributed) or just the *p* value (if data are not normally distributed)

	**Correlation of NFL *vs* NSE**	**Correlation of NFL *vs* S100B**	**Correlation of NSE *vs* S100B**
SS	*p* = 0.8397	*p* = 0.2972	*R* ^2^ = 0.3337 *p* = 0.2298
ES	*p* = 0.5580	*p* = 0.7506	*R* ^2^ = 0.0023 *p* = 0.8716
ST	*p* = 0.3710	*p* = 0.3509	*p* = 0.3570
ET	*R* ^2^ = 0.0118 *p* = 0.6893	*p* = 0.7827	*p* = 0.2782

### NFL concentrations are correlated with BBB disruption

**Figure 5.**
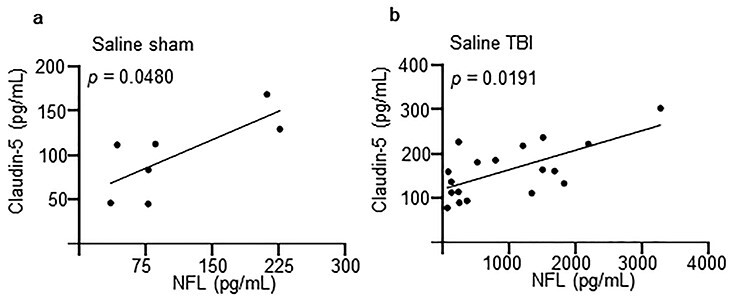
Neurofilament light (NFL) levels are correlated with blood–brain barrier disruption post traumatic brain injury. Correlation analysis was performed between the concentration of NFL, neuron-specific enolase (NSE), S100 beta (S100B) and claudin-5 3 hours post-traumatic brain injury (TBI). Four treatment groups were used: saline–sham (SS), ethanol–sham (ES), saline–TBI (ST) and ethanol–TBI (ET). (**a**) Correlation of NFL and claudin-5 levels were significant in the SS group (*p* = 0.0480). (**b**) Correlation of NFL and claudin-5 were significant in the ST group (*p* = 0.0191). The other treatment groups (ES and ET) did not show this correlation. (ES: *p* = 0.1469; ET: *R*^2^ = 0.0136; *p* = 0.6785). Overall slope comparison revealed significant differences in the treatment groups between NFL and claudin-5 (*F* = 3.157; *p* = 0.0320). *Post hoc* analysis revealed a significant difference in the slopes between the ST and ET group (*p* = 0.0303). Correlation with linear regression is shown. Group sizes: SS, n = 8; ES, n = 14; ST, n = 24; ET, n = 17. ^*^p < 0.05, ^**^p < 0.01, ^***^p < 0.001, ^****^p < 0.0001

Finally, we performed an exploratory analysis of the dataset to investigate any correlation between vascular, neuronal and glial damage markers (with or without concomitant EI) that may point to an effect of EI on the transport of the marker proteins from the brain parenchyma to the bloodstream. Taken alone, the correlations between NSE, NFL and S100B were poor when their values in individual mice were considered, although ANCOVA revealed significant differences between the slopes of NFL/S100B (*F* = 13.41; *p* < 0.0001) and NSE/S100B (*F* = 13.11; *p* < 0.0001), but not between the slopes of NFL/NSE (*F* = 0.4236; *p* = 0.7368). Notably, the values of blood ethanol, NSE, NFL and S100B concentrations displayed substantial variability despite the use of inbred mouse lines and standardized equipment. Surprisingly, the levels of the 3 markers in individual mice (distinct according to the treatment group) displayed poor correlation (NFL *vs* NSE; SS: *p* = 0.8397, ES: *p* = 0.5580, ST: *p* = 0.371, ET: *R*^2^ = 0.0118; *p* = 0.6893; NFL *vs* S100B; SS: *p* = 0.2972, ES: *p* = 0.7506, ST: *p* = 0.3509, ET: *p* = 0.7827; NSE *vs* S100B; SS: *R*^2^ = 0.3337; *p* = 0.2298, ES: *R*^2^ = 0.0027 *p* = 0.8716, ST: *p* = 0.3570, ET: *p* = 0.2782; Table 1). We also assessed the effect of EI on the plasma levels of the neuronal and glial injury biomarkers and the marker of vascular integrity. Interestingly, ANCOVA of NFL and claudin-5 values showed a significant difference between the slopes of NFL/claudin-5 (*F* = 3.157; *p* = 0.0320); *post hoc* analysis revealed significant correlation in the SS group (*p* = 0.0480; Figure 5a and Table 2) and in the ST group (*p* = 0.0191; Figure 5b and Table 2) but not in the other treatment groups (ES: *p* = 0.1469, ET: *R*^2^ = 0.0136; *p* = 0.6785; Table 2) and a significant difference between the slopes of the ST and ET group (*p* = 0.0303). However, in the case of NSE and claudin-5, ANCOVA revealed no significant differences between the slopes of NSE/claudin-5 across treatment groups (*F* = 0.9541; *p* = 0.4207); correlation between the 2 markers was very poor in each of the 4 treatment groups (SS: *R*^2^ = 0.0379; *p* = 0.6757, ES: *R*^2^ = 0.0001; *p* = 0.9716, ST: *p* = 0.8060, ET: *R*^2^ = 0.1597; *p* = 0.1251; Table 2). Lastly, when correlating claudin-5 with S100B, the ANCOVA showed a significant difference between the slopes of S100B/claudin-5 (*F* = 3.905; *p* = 0.0151); *post hoc* analysis revealed no significant differences within each of the treatment groups (SS: *R*^2^ = 0.0819; *p* = 0.5825, ES: *R*^2^ = 0.0993; *p* = 0.3453, ST: *p* = 0.6053, ET: *p* = 0.7892), but a significant difference between the slopes of the ST and ET groups (*p* = 0.0201) and between the slopes of the ET and ES groups (*p* = 0.0347). Thus, the severity of vascular disruption correlates with NFL concentrations, but this is not true for NSE and S100B.

**Table 2:** Plasma neurofilament light (NFL) concentrations are correlated to claudin-5 concentrations post traumatic brain injury. Correlation analysis was performed between the concentration of NFL, neuron-specific enolase (NSE), S100 beta (S100B) and claudin-5 3 hours post-traumatic brain injury (TBI) and ethanol TBI, 4 treatment groups were used: saline–sham (SS), ethanol–sham (ES), saline–TBI (ST) and ethanol–TBI (ET). Correlation of NFL and claudin-5 levels were significant in the SS group (*p* = 0.0480) and in the ST group (*p* = 0.0191) but not in the other treatment groups (ES: *p* = 0.1469, ET: *R*^2^ = 0.0136; *p* = 0.6785). Slope comparison revealed significant differences between the slopes in NFL and claudin-5 (*F* = 3.157; *p* = 0.0320). *Post hoc* analysis revealed a significant difference in the slopes between the ST and ET groups (*p* = 0.0303). Correlation of NSE and claudin-5 levels was very poor in each of the 4 treatment groups (SS: *R*^2^ = 0.0379; *p* = 0.6757, ES: *R*^2^ = 0.0001; *p* = 0.9716, ST: *p* = 0.8060, ET: *R*^2^ = 0.1597; *p* = 0.1251). Overall slope comparison revealed no significant differences between the slopes in NSE and claudin-5 (*F* = 0.9541; *p* = 0.4207). Correlation of S100B and claudin-5 were very poor within the single treatment groups (SS: *R*^2^ = 0.0819; *p* = 0.5825, ES: *R*^2^ = 0.0993; *p* = 0.3453, ST: *p* = 0.6053, ET: *p* = 0.7892). Overall slope comparison revealed significant differences between the slopes in S100B and claudin-5 (*F* = 3.905; *p* = 0.0151). *Post hoc* analysis revealed a significant difference between the slopes of ES and ET (*p* = 0.0347) and ST and ET (*p* = 0.0201). Correlation tables are shown, with both *R*^2^ and *p* values (if data are normally distributed) or just the *p* value (if data are not normally distributed)

	**Correlation NFL *vs* claudin-5**	**Correlation NSE *vs* claudin-5**	**Correlation S100B *vs* claudin-5**
SS	***p*** **= 0.0480**	*R* ^2^ = 0.0379 *p* = 0.6757	*R* ^2^ = 0.0819 *p* = 0.5825
ES	*p* = 0.1469	*R* ^2^ = 0.0001 *p* = 0.9716	*R* ^2^ = 0.0993 *p* = 0.3453
ST	***p* = 0.0191**	*p* = 0.8060	*p* = 0.6053
ET	*R* ^2^ = 0.0136 *p* = 0.6785	*R* ^2^ = 0.1597 *p* = 0.1251	*p* = 0.7892

## Discussion

The goal of the present study was to determine if any effect of ethanol could be detected on the plasma levels of 4 biomarkers of brain damage after TBI. We aimed to provide initial, preclinical evidence to eventually inform future studies in humans, and also to provide evidence for the use of plasma biomarkers in the study of the effect of comorbidities on TBI pathogenic processes in preclinical models. We demonstrated that TBI induces the elevation of peripheral-damage-related neuronal, glial and vascular biomarkers (NSE, NFL, S100B and claudin-5) and that ethanol appears to decrease NSE but not NFL, S100B or claudin-5 concentrations in a dose-dependent manner in plasma samples. EI alone increases S100B, but not NFL, NSE or claudin-5, levels. Interestingly, even in the absence of ethanol modulation, NFL plasma concentrations correlated with the concentrations of claudin-5, but not with NSE or S100B concentrations. Thus, our data suggest that (1) EI reduces NSE concentrations, but not necessarily the overall acute burden of neurodegeneration and glial damage as explored by NFL and S100B; (2) ethanol alone is able to increase S100B levels; and (3) concentrations of NFL, but not of NSE or S100B, correlate with elevation of the vascular disruption marker. Although it has been reported in the literature that brain and spinal cord injury results in systemic inflammation and disturbed hepatic metabolism [46–48], we have shown that TBI does not affect the ethanol concentration in plasma, suggesting that hepatic ethanol metabolism does not seem to be disturbed in our specific conditions.

Both NSE and NFL have been extensively investigated as markers of neuronal damage in TBI and display an overall significant elevation in brain trauma [1, 4, 5, 49–54]. Although NSE was originally identified as an oncomarker [55], elevation in trauma patients may be affected as a consequence of hemolysis and multiple traumas without head injury, and in rats with ischemic injury to abdominal organs [56] (no report of false-positive NSE elevation due to lung cancer has ever been reported). However, in our experimental model, only a TBI was administered and NSE can therefore be used as a marker of neuronal damage [57]. NFL, on the other hand, has been more recently developed in preclinical models of trauma [16] and has great potential as a TBI prognostic marker (although NFL is elevated in essentially all conditions affecting the central and peripheral nervous systems) [58–61]. It has been hypothesized that the 2 markers may not necessarily reflect the same pathophysiological processes in TBI [1, 62] as they display different kinetics (NSE elevation peaks early after TBI, NFL levels keep increasing over several days [1]) and distinct cellular localization. NFL is highly enriched in large, myelinated axons [63, 64], whereas NSE is an abundant soluble cytoplasmic protein [65]. Our data show that ethanol levels are inversely correlated with the former but not the latter. Previous preclinical studies (related to the plasma samples analysed in this work) have shown that EI prior to TBI is associated with enhanced neuronal survival, improved network activity, improved behavioral performance and reduced neuroinflammatory responses [29–32]. Thus, within the hypothesis that NSE and NFL are markers of different types of neuronal damage, our data imply that EI is not effective at protecting axonal integrity but may have an effect on survival of neurons (in agreement with the histological data cited above showing reduced loss of cell bodies in TBI with concurrent EI [13, 30]). Likewise, ethanol appears ineffective at reducing TBI-induced S100B elevation (in agreement with previous data showing no effect of EI on Glial fibrillary acidic protein (GFAP) elevation upon trauma [31]) but it is sufficient to induce S100B elevation per se, as previously reported in human cohorts [20]. Thus, the clinical interpretation of plasma biomarker levels in cases of concurrent EI should be cautious and involve multiple analytes, since S100B levels may be falsely elevated because of ethanol itself (or because of additional conditions such as neoplastic disorders, e.g. [66]) and NSE levels may be reduced because of a protective effect of ethanol. In this context, NFL levels appear the least sensitive to ethanol and may be considered as the most reliable indicator of damage. Since it has been recently reported that EI appears to be protective, especially in patients with penetrating brain injuries [67], it would be interesting to investigate whether a differential effect of EI on biomarkers (NSE *vs* NFL) may appear in injuries with more or less extensive axonal damage versus neuronal damage, as suggested by our findings.

Claudin-5 is a tight junction protein that is highly expressed in endothelial cells, where it contributes to the integrity of the BBB [68]. Plasma levels of claudin-5 have been proposed as a marker of vascular disruption in multiple conditions involving the intestine, liver or vasculature [69–71] and, in conditions in which the damage is limited to the brain, have been proposed a marker of BBB integrity [72–74]. Although systemic inflammation may possibly contribute to claudin-5 levels, in our model, TBI was the only substantial injury administered to mice. Taken together with published data on other brain injury models that have used claudin-5 as a marker for BBB disruption [72–75], it would appear reasonable to assume that the majority of claudin-5 would derive from the injured brain. Although ethanol appears to decrease the inflammatory response induced by TBI [29], it does not affect the peripheral elevation in soluble claudin-5 triggered by TBI. This finding is in agreement with a previous report of the lack of an effect of ethanol on the downregulation of interleukin-25 (IL-25), a central regulator of BBB integrity, upon trauma [32].

Interestingly, claudin-5 concentrations directly correlated with NFL concentrations, but not with NSE and S100B concentrations, upon trauma (with or without ethanol). This finding suggests that while the appearance of NFL in plasma is facilitated by the increase in permeability of the BBB, plasma NSE and S100B levels increase irrespective of BBB integrity. In fact, clearance of NSE from the central nervous system (CNS) is only mildly related to BBB markers in TBI patients [76]. It is tempting to speculate that other systems, such as the recently identified glymphatic system, may be involved in the BBB-independent transfer of NSE from the brain to the blood. This finding further underscores how peripheral biomarkers may explore distinct aspects of the pathophysiology of TBI.

As a potential limitation of this study, it is worth noting that we used a relatively small number of biomarkers to investigate the effect of ethanol on TBI prognosis. Various biomarkers might display distinct responses to ethanol in TBI, like inflammatory mediators [77] or astrocyte-enriched proteins [78]. The small number of biomarkers explored was due to the comparatively small volume of plasma recovered from each mouse and the volume required for ELISAs. In the future, a more widespread use of high-sensitivity SIMOA systems will allow in-depth exploration of biomarkers of TBI in murine models. In addition to the amount of samples, the sensitivity of the assay poses another limitation. The SIMOA technology is much more sensitive than an ordinary ELISA (pg/ml for ELISA, fg/ml for SIMOA [79, 80]). A third limitation may be the exploratory, retrospective design of this study; although operators and procedures were similar across several studies, unforeseen biases may contribute to the large variability of the NSE and NFL values. It must also be considered that the experimental TBI model employed was titrated to obtain a detectable injury (in terms of behavioral and histological readouts [30, 31]) without massive tissue damage, bone fracture or parenchymal hematomas. This approach aims to reduce unnecessary suffering of the experimental animals and limit experimental variability. As a consequence, these findings must be discussed within the limitation of the severity of the TBI and may not necessarily apply to more severe conditions with large hematomas. A fourth limitation is the inclusion of male mice and a single dose of ethanol. The present study represents a model for a drink-and-drive trauma model, which is the most common occurrence in young patients and disproportionately affects men; nevertheless, gender differences in trauma response are important and should be addressed in a dedicated study. A final limitation may be the single time point taken for this study; it would be of interest to explore the effect of acute ethanol on peripheral biomarkers over a longer time course. However, due to the exploratory nature of this study and the use of previously collected samples this was not possible. Despite these limitations, our data contribute to the ongoing debate on the interpretation of TBI biomarkers in conditions of EI [20, 81], indicating that plasma NFL levels may be the indicator of damage least affected by EI.

## Conclusions

Acute EI concomitant to TBI decreases plasma levels of NSE (compared to saline-treated mice) but not of NFL, claudin-5 or S100B; notably, ethanol alone is sufficient to elevate plasma S100B levels. Therefore, the neuroprotective effect of EI may be restricted to acute cellular damage rather than axonal damage. Our preclinical data suggest that EI may influence some, but not all, biomarker levels in TBI patients and this effect may have to be taken into account in future studies of clinical cohorts.

## Abbreviations

24-G: 24-Gauge; ANCOVA: analysis of covariance; ANOVA: analysis of variance; BAC: blood alcohol concentration; BBB: blood–brain barrier; BSA: bovine serum albumin; CNS: central nervous system; EDTA: Ethylenediaminetetraacetic acid; EI: ethanol intoxication; ELISA: enzyme-linked immunosorbent assay; ES: ethanol–sham group; ET: ethanol–TBI group; GFAP: Glial fibrillary acidic protein; IL-25: interleukin-25; NeuN: neuronal nuclear protein; NFL: neurofilament light; NSE: neuron-specific enolase; OD: optical density; PBS: phosphate buffered saline; PFA: paraformaldehyde; RLU: relative light units; RT: room temperature; S100B: S100 beta; SIMOA: single-molecule array; SS: saline–sham group; ST: saline–TBI group; TBI: traumatic brain injury.
